# Altered Spectrum of Lymphoid Neoplasms in a Single-Center Cohort of Common Variable Immunodeficiency with Immune Dysregulation

**DOI:** 10.1007/s10875-021-01016-4

**Published:** 2021-04-19

**Authors:** Claudia Wehr, Leonora Houet, Susanne Unger, Gerhard Kindle, Sigune Goldacker, Bodo Grimbacher, Andrés Caballero Garcia de Oteyza, Reinhard Marks, Dietmar Pfeifer, Alexandra Nieters, Michele Proietti, Klaus Warnatz, Annette Schmitt-Graeff

**Affiliations:** 1 Department of Medicine I, Medical Center – University of Freiburg, Faculty of Medicine, University of Freiburg, Freiburg, Germany; 2 Center for Chronic Immunodeficiency, Medical Center - University of Freiburg, Faculty of Medicine, University of Freiburg, Freiburg, Germany; 3 Institute for Immunodeficiency, Center for Chronic Immunodeficiency (CCI), Medical Center - University of Freiburg, Faculty of Medicine, University of Freiburg, Freiburg, Germany; 4 FREEZE Biobank, Center for Biobanking, Medical Center and Faculty of Medicine, University of Freiburg, Freiburg, Germany; 5 Division of Immunodeficiency, Department of Rheumatology and Clinical Immunology, Medical Center - University of Freiburg, Faculty of Medicine, University of Freiburg, Breisacher Str. 115, 79106 Freiburg, Germany; 6 DZIF – German Center for Infection Research, Satellite Center Freiburg, Freiburg, Germany; 7 CIBSS – Centre for Integrative Biological Signalling Studies, Albert-Ludwigs University, Freiburg, Germany; 8 RESIST – Cluster of Excellence 2155 to Hanover Medical School, Satellite Center Freiburg, Freiburg, Germany; 9 University of Freiburg, Freiburg, Germany

**Keywords:** Common variable immunodeficiency (CVID), lymphoma, Hodgkin lymphoma (HL), diffuse large B cell lymphoma (DLBCL), marginal zone lymphoma (MZL), *CTLA4*

## Abstract

**Purpose:**

Common variable immune deficiency (CVID) confers an increased risk of lymphoid neoplasms, but reports describing the precise WHO specification of the lymphoma subtypes and their immunological environment are lacking. We therefore classified lymphomas—occurring in a cohort of 21 adult CVID patients during a 17-year period at our center—according to the 2016 WHO classification and characterized the local and systemic immunological context

**Results:**

The median time between the onset of CVID and lymphoma was 14 years. Patients showed a high prevalence of preceding immune dysregulation: lymphadenopathy (*n* = 13, 62%), splenomegaly (*n* = 18, 86%), autoimmune cytopenia (*n* = 14, 67%), and gastrointestinal involvement (*n* = 15, 71%). The entities comprised extranodal marginal zone lymphoma (*n* = 6), diffuse large B cell lymphoma (*n* = 7), plasmablastic lymphoma (*n* = 1), classic Hodgkin lymphoma (*n* = 4, including three cases with germline *CTLA*4 mutations), T cell large granular lymphocytic leukemia (*n* = 2), and peripheral T cell lymphoma, not otherwise specified (*n* = 1), but no follicular lymphoma. An Epstein-Barr virus association was documented in eight of 16 investigated lymphomas. High expression of PDL1 by tumor cells in five and of PDL1 and PD1 by tumor-infiltrating macrophages and T cells in 12 of 12 investigated lymphomas suggested a tolerogenic immunological tumor environment.

**Conclusion:**

In summary, a diverse combination of specific factors like genetic background, chronic immune activation, viral trigger, and impaired immune surveillance contributes to the observed spectrum of lymphomas in CVID. In the future, targeted therapies, e.g., PD1/PDL1 inhibitors in CVID associated lymphomas with a tolerogenic environment may improve therapy outcome.

**Supplementary Information:**

The online version contains supplementary material available at 10.1007/s10875-021-01016-4.

## Introduction

Morbidity and mortality of CVID patients under immunoglobulin replacement are mainly determined by malignancies—lymphoid neoplasms and gastric adenocarcinoma—and clinical manifestations of immune dysregulation rather than infections [1–3]. The predisposition of CVID patients to develop lymphoid neoplasms has long been recognized [1, 2, 4–11]. Similar to other centers [8, 12, 13], 4% of our CVID patients developed lymphoma [14]. The timely detection and accurate diagnosis of a lymphoid neoplasm in CVID patients are both clinically and pathologically challenging. This can be due to pre-existing and concomitant lymphoid hyperplasia (long-standing lymphadenopathy, splenomegaly) and lack of biomarkers hamper to set the optimal time point for a biopsy. Additionally, the diagnosis of an overt malignant lymphoma and the assignment to a specific disease entity can pose significant challenges to the pathologist [15, 16]. A precise subclassification of immunodeficiency-associated lymphomas following the updated 2016 WHO system can be difficult as it was established for immunocompetent patients and immunodeficiency-associated lymphomas may present with pathologic variants. [17]. Boundaries between non-malignant lymphoproliferative disorders (LPD) and overt lymphomas are often difficult to recognize [18, 19]. Non-neoplastic LPDs may present with a profound modulation of the innate lymph node structure and contain morphologically abnormal lymphoid populations, including blast cells, especially in Epstein-Barr virus (EBV)-positive cases. On the other hand, abundant reactive immune infiltrates in the microenvironment of lymphomas can lead to misdiagnosis as a non-neoplastic lesion. Pitfalls also arise from an inadequate procurement of specimens, especially when samples are taken from the concomitant LPD and not from the neoplastic process. A multiparameter approach integrating routine and ancillary techniques as well as clinical information are mandatory for an appropriate diagnosis.

The mechanisms of lymphomagenesis in CVID are not completely understood. Higher IgM levels at diagnosis of CVID, female sex [1], a phenotype of late-onset combined immunodeficiency (loCID) [20], polyclonal LPD [2, 12], and immune thrombocytopenia [13] have been associated with a higher risk in some cohorts; the latter however is not yet confirmed in the recent US registry report [12]. While increased risk for lymphoma development in CVID is broadly recognized, a precise categorization following the current WHO classification is only given for a subset of published lymphomas [11]. For a relevant number of cases, only a lineage assignment but no further definitive WHO classification is provided. Most publications report lymphomas of B cell origin, while T cell lymphomas are rare. Among the defined entities, the literature describes a predominance of extranodal marginal zone lymphoma (ENMZL) arising in the mucosa-associated lymphoid tissue (MALT) of the gastrointestinal tract, the salivary glands or in the bronchus-associated lymphoid tissue [4, 5]. But also classic Hodgkin lymphoma (CHL), diffuse large B cell lymphoma (DLBCL), and rare T cell neoplasms such as T cell large granular lymphocytic leukemia (T-LGLL) have been described [1, 2, 8, 20–22].

Thus, the characterization of the spectrum of CVID-associated lymphoid neoplasms and the biologic factors involved in their development warrant additional studies. To approach these questions, we retrospectively reviewed the clinical presentation, immunological phenotype, histologic features, and—if available—molecular abnormalities of 21 CVID-associated lymphoma cases collected from our institution.

## Methods

### Patient Cohort and Data Collection

Patients with CVID and histopathologically established diagnosis of lymphoid neoplasm were retrospectively identified. The diagnosis of CVID was based on ESID/PAGID criteria [23]. Patients with possible secondary immunodeficiency due to lymphoma or a concurrent diagnosis (<1 year between diagnosis of CVID and lymphoma) were excluded. The institutional review board approved the study according to the Declaration of Helsinki (No: 239/1999 and 121/11).

### Targeted Next-Generation Sequencing of Germline Variants

Germline whole-exome sequencing (WES) data was available for 12 patients; one patient underwent targeted sequencing of the *CTLA4* locus (Supplemental Methods).

### Histopathological, Immunohistological, and Molecular Analyses of Tissue Specimens

The diagnosis of lymphoid neoplasm was based on the histopathological evaluation of tissue specimens and supported by conventional immunohistochemistry and molecular methods (Supplemental Methods). The large majority of cases were reevaluated according to the guidelines of the current revised 2016 WHO classification [16]. We further defined all tumor samples according to the unifying nomenclature for immunodeficiency-associated LPD [15]. DNA extracted from formalin-fixed paraffin-embedded tissue was available in six lymphomas and analyzed on an Illumina TruSight Lymphoid Panel. Of the six samples, four were rejected due to low sample quality.

## Results

### Characteristics of the Patient Cohort

We identified 21 CVID patients with lymphoma (Table 1). Germline genetic disease-associated variants were proven in five of 13 analyzed patients: three *CTLA4*, one *BACH2*, and one *TNFSR13B*. Among 162 patients in the CVID cohort at our center who underwent genetic testing, 7.4% (*n* = 12) carried rare C*TLA4* variants, 16% (*n* = 26) carried rare *TNFSR13B* variants, and 4.9% (*n* = 8) rare *BACH2* variants. The median age of onset of first symptoms attributed to CVID was 29.5 years (interquartile range [IQR]: 21–38 years). This appeared lower in the subgroup with *CTLA4* mutations (11, 15, 16 years). The median age at diagnosis of lymphoma was 38 years (IQR: 32–51 years). The median time between the onset of first symptoms attributed to CVID and diagnosis of lymphoid malignancy was 14 years (IQR 8–17 years); this was comparable in the subgroup of patients with *CTLA4* mutations (15, 17, 18 years). Men were overrepresented in our cohort (male *n* = 15, female *n* = 7) consistent with the increased risk of lymphoma development in men in the general population [24].

**Table 1:** Clinical characteristics of CVID patients with lymphoid neoplasms.

Patient	sex	IEI	Genetics (result)	MOI	ACMG	age at onset of symptoms of CVID	age at diagnosis of lymphoid neoplasm	lymphadenopathy	splenomegaly	inflammatory lung involvement	autoimmune cytopenia (ITP/AIHA/AIN)	GI involvement	other autoimmunity	immunosuppression before onset of lymphoid neoplasm	others	Duration of follow-up after diagnosis of lymphoid neplasm until death or last visit
1	m	CVID	BACH2, c.2362G>A p.Glu788Lys	AD	BS1, BS2 strong, PP5 supporting	42	48	1	1	0	1	lymphocytic colitis	AION	steroid		8 years
2	f	CVID	-			42	52	0	1		1	lymphocytic duodenitis, colitis	0	steroid		1 month
3	m	CVID	-			35	49	1	1	0	1	0	0	steroid		3 years
4	f	CVID	-			45	48	1	1	0	1	diarrhea and wasting (histology not available)	neurodermitis, diabetes mellitus type I, autoimmune hepatitis	steroid, azathioprine		5 months
5	f	CVID, evolved into loCID	-			38	49	1	1	0	1	seronegative enteropathy with features of refractory celiac disease and nodular lymphofollicular hyperplasia	nodular regenerative hyperplasia of the liver	steroid, Rituximab		2 years 1 month
6	m	CVID with CTLA4 mut.	CTLA 4, c.531_544del, pF179fs	AD	PVS1 very strong, PM2 moderate, PP3 supporting	16	33	1	1	0	1	duodenal nodular lymphatic hyperpalsia, lymphocytic ileitis	lymphocytic encephalomyelitis	steroid, azathioprine		7 years 5 months
7	m	CVID	TNFRSF13B, c.542C>A, p.A181E	AD AR	BS1, BS2 strong, BP1, BP4 supporting, PM1 moderate, PM5, PP5 supporting	30	46	0	1	0	1	0	0	azathioprine	adenocarcinoma stomach	23 years
8	m	CVID	-			31	32	1	1	0	1	seronegative enteropathy with features of refractory celiac disease and lymphofollicular hpyerplasia	0	0		13 years
9	m	CVID	NFKB2, c.2072-3C>T, intronic	AD	BS1, BS2 strong, BP4, BP6 supporting	26	38	1	1	0	0	0	0	0		14 years 8 months
10	m	CVID evolved into loCID	-			21	33	0	1	1	0ulcerative colitis-like enteropathy	0	steroid, mesalazin	recurrent CMV		5 years
11	m	CVID	-			37	38	1	1	0	0	0	thyroiditis	0		15 years
12	m	CVID with CTLA4 mut.	CTLA4 c.223C>T, p.R75W	AD	PM1, PM2, PP5 moderate, PP2, PP3 supporting	11	28	1	1	1	1	seronegative enteropathy with features of refractory celiac disease	interstitial nephritis	steroid, Rituximab		2 years 8 months
13	m	CVID (loCID)	no rare variants in PID associated genes identified			9	29	1	1	0	1	seronegative enteropathy with features of refractory celiac disease	Diabetes mellitus Type I, autoimmune hepatitis	steroid	CMV colitis	7 months
14	f	CVID	-			21	80	0	0	0	1	0	0	0		2 months
15	f		CVID STAT1 c.796G>A; p.V266I	AD AR	BS1, BS2 strong, BP4, PP2 supporting	47	52	1	1	1	0	nodular lymphofollicular hyperplasia (ileum)	0	steroid		2 years 2 months
16	m	CVID, evolved into	loCIDno rare variants in PID associated genes identified			27	47	0	1	1	0	duodenitis, ileitis, cryptitis, chronic Campylobacter infection	nodular regenerative hyperplasia of the liver, arthritis	steroid, sulfasalazin, sirolimus		1 year 8 months
17	f	CVID (loCID)	no rare variants in PID associated genes identified			44	52	0	1	1	1	ileitis, colitis (ulcerative colitislike)	nodular regenerative hyperplasia of the liver	steroid		12 years
18	m	CVID	no rare variants in PID associated genes identified			27	46	1	1	0	0	chronic diarrhea and wasting (histology not available)	reactive arthritis	sulfasalazin, leflunomid, hydrocychloroquine, steroid		9 years 2 months
19	m	CVID, evolved into loCID	no rare variants in PID associated genes identified			38	52	0	1	0	0	nodular lymphofollicular hyperplasia (ileum), Giardia lamblia infection	0	steroid	squamous cell carcinoma	5 years 7 months
20	f	CVID with CTLA4 mut.	CTLA4 c.105C>A, p.C35*	AD	PVS1 very strong, PP5 strong, PM2 moderate, PP3 supporting	15	30	0	0	1	1	seronegative enteropathy with features of refractory celiac disease	arthritis, CNS inflammation, psoriasis	steroid		3 months
21	m	CVID	no rare variants in PID associated genes identified			17	34	1	0	0	1	0	0	steroid		2 years

Abbreviations: IEI: inborn error of immunity, CVID: common variable immunodeficiency, loCID: late-onset combined immunodeficiency, NGS: next-generation sequencing, MOI: mode of inheritance, ITP: idiopathic thrombocytopenic purpura, AIHA: atuoimmune hemolytic anemia, AIN: autoimmune neutropenia, GI involvement: gastrointestinal invovlement, EBV:Ebstein-Barr virus, AION: Anterior Ischemic Optic Neuropathy, CTX: chemotherapy, PD: progressive disease, HCT: hematopoietic cell transplantation, CMV: cytomegalo virus. 1: present, 0: absent, n.d.: not done

### High Prevalence of Immune Dysregulation and Immunosuppressive Treatment Preceding the Development of Lymphoid Neoplasm in CVID Patients

**Fig. 1:**
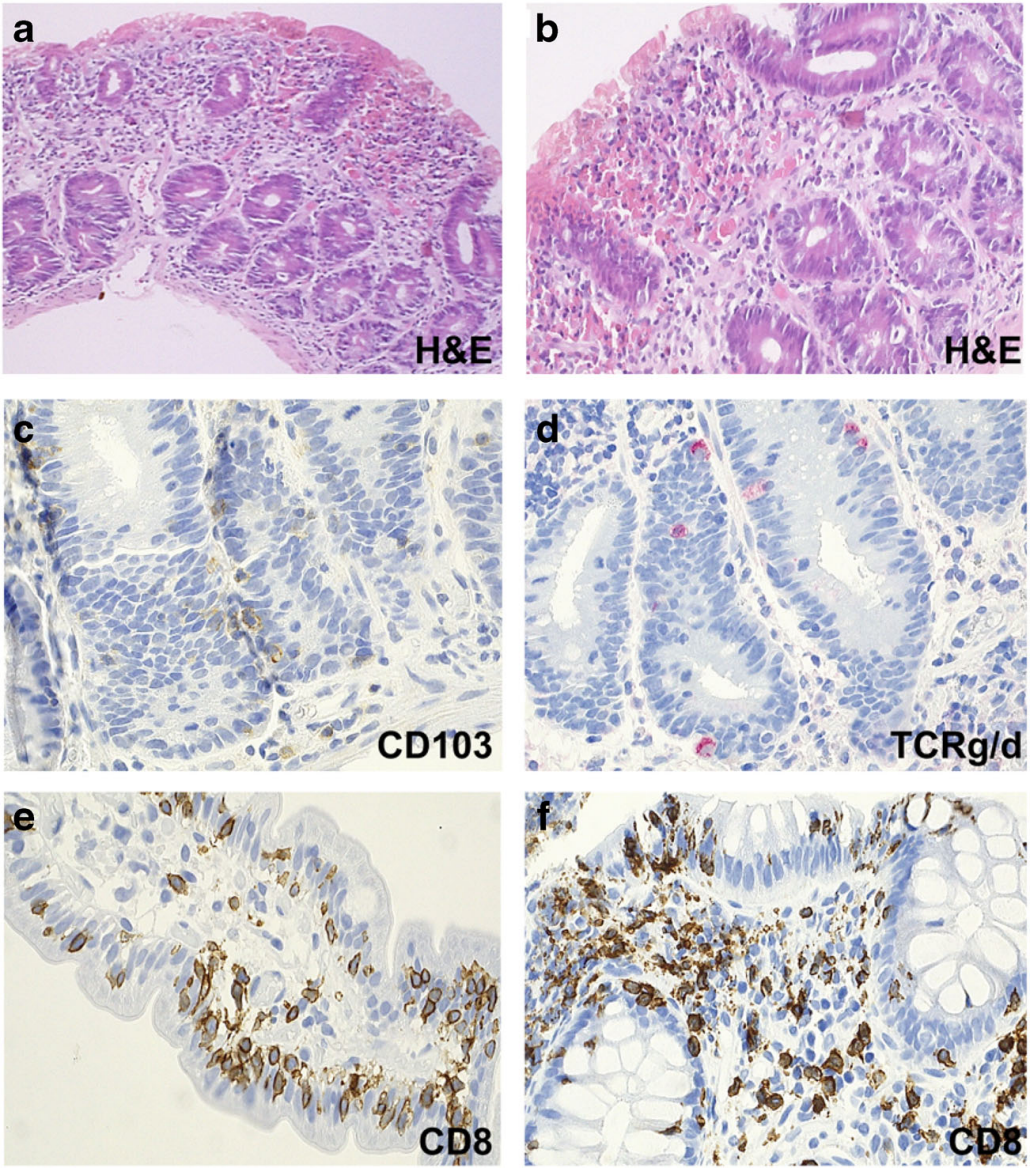
*Histology of gastrointestinal biopsies of CVID patients with lymphoma* (**a**–**d**) Refractory celiac disease type 1-like enteropathy (patient 05). (**a**–**b**) Complete flattening of the duodenal mucosa with loss of villi and damage of the surface epithelium. (**c**–**d**) Most Intraepithelial lymphocytes belonged to a CD103-positive population of gut lymphocytes (**d**), expressed CD8 and cytotoxic molecules such as TIA but were CD56 negative (not shown). Some TCRγδ+ T cells were present (**d**). (**e**-**f**) Microscopic enteritis: non-atrophic enteropathy with normal villous:crypt ratio but an increase in intraepithelial CD8+ T-cells (patient 06)

In the general CVID cohort, approximately one-third of CVID patients have non-infectious disease-related complications [2, 20, 25, 26]; however, in our lymphoma cohort, all patients suffered from preceding non-infectious disease-related complications. Consistent with the literature [2, 12], preceding lymphadenopathy (*n* = 13, 62%) or splenomegaly (*n* = 18, 86%) was highly prevalent in our cohort. Additionally, both autoimmune cytopenias (*n* = 14, 67%) and non-infectious gastrointestinal involvement (*n* = 15, 71%) were more prevalent in CVID patients developing lymphoma compared to the general CVID population (30% and 16%, respectively) [14]. Upon reevaluation of intestinal biopsies of CVID patients with lymphoma, we observed a variety of morphologic changes including ulcerative colitis-like colitis, celiac-like enteropathy, or microscopic enteritis with oligoclonal T cell expansion (Fig. 1). Due to the high prevalence of non-infectious inflammatory complications, 17 patients (81%) had received systemic immunosuppressive therapy before the diagnosis of lymphoma. Immunoglobulin levels and lymphocyte subpopulations were collected at diagnosis of CVID and before first lymphoma treatment. Even when accounting for 30% of missing values, the data indicated that the majority of studied patients (76%) had low IgM levels at diagnosis of CVID which was in contrast to previously published data [1, 2], and CD4 lymphocytopenia was more prevalent at diagnosis of lymphoma (*n* = 11, 52%) compared to initial diagnosis of CVID (*n* = 6, 29%). It remains an open question, whether the immunosuppressive treatment, the lymphoid neoplasm, the natural course of the immunodeficiency disorder, or a combination of these underlie the development of CD4 lymphocytopenia.

### Detailed Characterization of Lymphoid Neoplasms

The following entities were diagnosed (Table 2): ENMZL (*n* = 5, 24%), splenic MZL (*n* = 1, 5%), DLBCL (*n* = 7, 33%), plasmablastic lymphoma (PBL, *n* = 1, 5%), mixed cellularity CHL (MCCHL, *n* = 4, 19%), T-LGLL (*n* = 2, 10%), and nodal peripheral T cell lymphoma, not otherwise specified (PTCL, NOS, *n* = 1, 5%). According to the recently proposed unifying nomenclature for immunodeficiency-associated LPD [15], six cases were assigned to the category indolent B cell lymphoma while eight cases fulfilled criteria of aggressive B cell lymphoma. The lymphoid neoplasm was EBV associated in eight of 16 cases (50%), while an additional three cases (19%) only contained a low amount of small EBV+ bystander cells (Supplemental Table 1).

**Table 2:** Key pathological findings in CVID patients with lymphoid neoplasms.

Patient	Classification of lymphoid neoplasm according to WHO classification 2016*	Hans classifier	EBV status lymphoma	Assessment of clonality status #
1	EBV+ DLBCL	non-GCB	+	clonal IgH gene rearrangement
2	EBV+ DLBCL	non-GCB	+	clonal IgH gene rearrangement
3	splenic MZL		-	clonal IgH gene rearrangement
4	DLBCL, NOS	non-GCB	-	clonal IgH gene rearrangement
5	DLBCL, NOS with preceding nodal MZL	non-GCB	-	clonal IgH gene rearrangement
6	MCCHL		+	clonal IgH gene rearrangement
7	extranodal MZL of MALT type (lung, stomach)		-	clonal IgH gene rearrangement
8	extranodal MZLof MALT type (lung)		-	clonal IgH gene rearrangement
9	EBV+ DLBCL	non-GCB	+	clonal IgH gene rearrangement
10	T-LGLL		unknown	clonal TCRgamma gene rearrangement
11	T-LGLL		unknown	clonal TCRgamma gene rearrangement
12	MCCHL		+	no rearranged IgH chain genes
13	plasmablastic lymphoma	non-GCB	+	clonal IgH gene rearrangement
14	PTCL, NOS		unknown	clonal TCRgamma gene rearrangement
15	extranodal MZL of MALT type (lung)		-	clonal IgH gene rearrangement.
16	extranodal MZL of MALT type (duodenum)		unknown	clonal IgH gene rearrangement
17	extranodal MZL of MALT type (lung)		unknown	clonal IgH gene rearrangement
18	MCCHL		+	no rearranged IgH chain genes
19	DLBCL, NOS (duodenum)	GCB	-	clonal IgH gene rearrangement
20	MCCHL		+	no rearranged IgH chain genes
21	DLBCL, NOS with preceding nodular lymphocyte predominant HD	GCB	-	clonal IgH gene rearrangement

#Assessment of clonality status by PCR using BIOMED-2 primers. Abbreviations: CVID: common variable immunodeficiency, EBV:Ebstein-Barr virus, DLBCL :diffuse large B-cell lymphoma, NOS: not otherwise specified, MCCHL: Mixed cellularity classic Hodgkin lymphoma, MZL marginal zone lymphoma, MALT mucosa-associated lympoid tissue, T-LGLL: T-cell large granular lymphocytic leukemia, PTCL: Peripheral T cell lymphoma, DLBCL: diffuse large B cell lymphoma, MZL: marginal zone lymphoma, MALT: mucosa-associated lymphatic tissue. 1: present, 0: absent, n.a.: not available

### Indolent B Cell Lymphomas

The six indolent B cell lymphomas included four ENMZL of pulmonary MALT, one ENMZL of duodenal MALT, and one splenic MZL.

### Extranodal marginal zone lymphoma of mucosa-associated lymphoid tissue

**Fig. 2:**
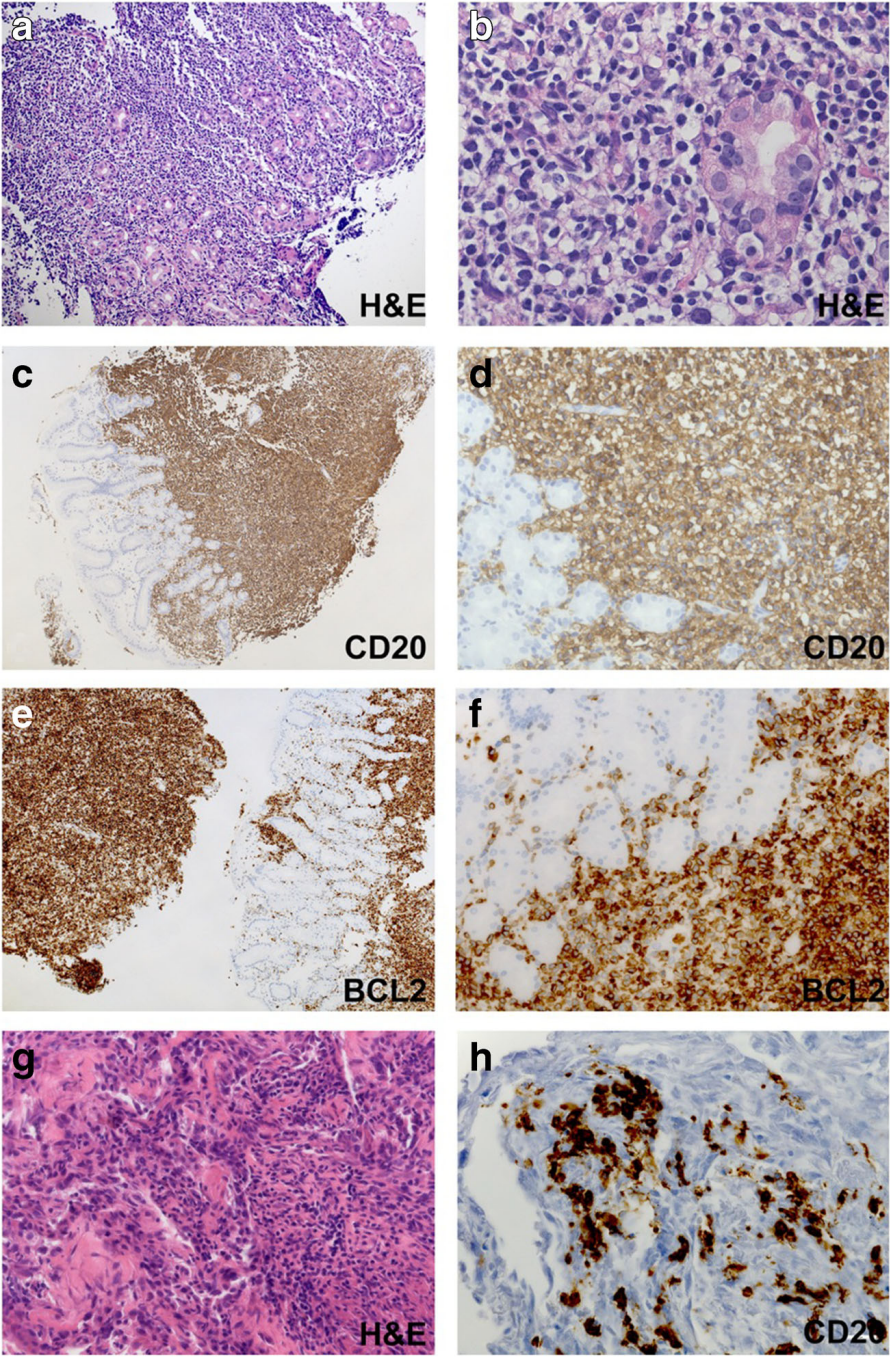
*Extranodal marginal zone lymphoma (ENMZL) of mucosa-associated lymphoid tissue (MALT) in lung and stomach.* Marginal zone lymphoma developing in MALT of stomach (**a**–**f**) and lung (**g**, **h**) (patient 07). Small to medium-sized CD20- (**c**, **d**) and BCL2 (**e**, **f**)-positive lymphocytes infiltrate the mucosa and submucosa of the stomach. Note the presence of lymphoepithelial lesions in B. The transbronchial biopsy shows crush artifact of the pulmonary parenchyma (**g**) and reveals CD20-positive B-cell infiltrates consistent with lung involvement by the MZL

Two patients presented with Helicobacter pylori-negative ENMZL of the MALT of the upper gastrointestinal tract (patient 08, 16): Patient 08 was diagnosed with MZL in the gastric mucosa 16 years after diagnosis and complete remission of a pulmonary ENMZL (Fig. 2a–f). Whether the gastric ENMZL was a relapse or a second neoplasm could not be evaluated. The biopsy contained rare EBER+ small lymphocytes. Patient 16 presented with ENMZL in the duodenum and showed, besides lymphoepithelial lesions, a marked plasma cell differentiation. EBV was not tested.

De novo pulmonary ENMZL was diagnosed in three patients (patient 08, 15, 17), all suffering from recurrent respiratory tract infections (Fig. 2g–h). Two specimens were tested for EBV but were negative.

### Splenic Marginal Zone Lymphoma (SMZL)

**Fig. 3:**
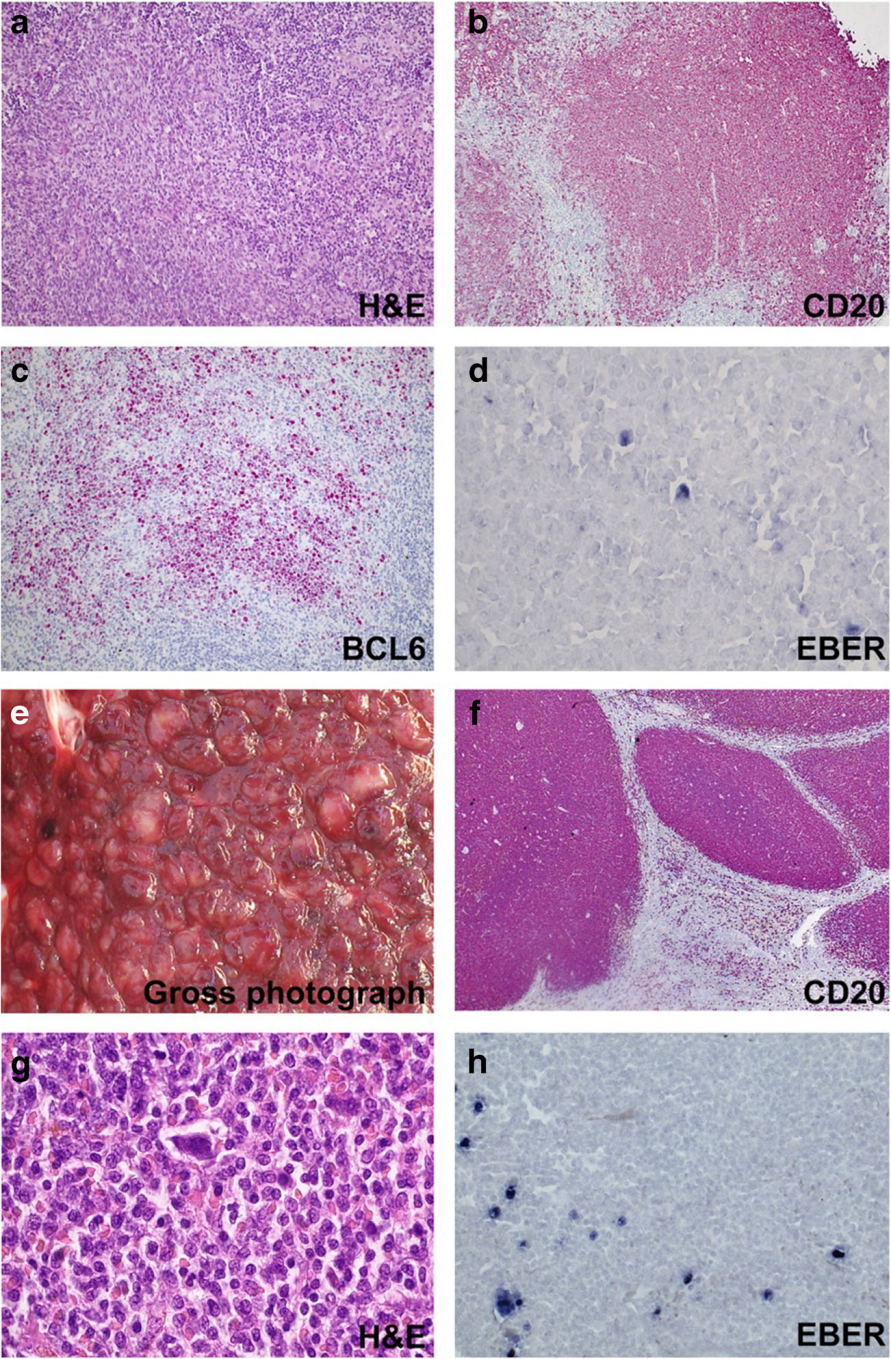
S*plenic marginal zone lymphoma preceded by early lesions of EBV lymphoproliferation.* Early lesions of EBV lymphoproliferation, follicular hyperplasia subtype (**a**–**d**) and subsequent splenic marginal zone lymphoma (**e**–**h**) (patient 03). Irregular expansion of germinal center cells (**a**) expressing CD20 (**b**) and BCL6 (**c**) in a lymph node harboring scattered EBER-positive lymphocytes (**d**). Splenectomy specimen (**e**–**h**) showing a nodular pattern both at gross examination of the cut surface (**e**) and on histologic slides (**f**) resulting from a proliferation of CD20-positive lymphocytes predominantly involving the white pulp (**g**). Predominantly small- to medium-sized lymphocytes with morphologic features of marginal zone cells surround scattered multinucleated cells reminiscent of Sternberg-Reed cells that coexist with rare small EBER-positive lymphocytes by CISH analysis (**h**)

Splenic MZL developed in patient 03 with preceding lymphoid hyperplasia and early lesions of EBV lymphoproliferation, follicular hyperplasia subtype (Fig. 3a–d). Splenectomy specimen showed a CD20+BCL2+IgM+IgD+ SMZL (Fig. 3e–f). A minority of small to large EBER+CD30+CD15− cells was present, predominantly associated with germinal centers (Fig. 3g–h) and was considered residual component of the preceding EBV lymphoproliferation [27]. The lymphoma was not classified as EBV + MZL as the dominant population was EBER negative.

In the MZL samples, tumor cells did not express PDL1, while moderate amounts of PDL1+ tumor-infiltrating cells (TICs), including macrophages and dendritic cells, and PD1+ tumor-infiltrating lymphocytes were present in the microenvironment (Fig. 6g–h, Supplemental Table 1).

### Aggressive B Cell Lymphoma

The category of aggressive B cell lymphomas included seven DLBCL (four DLBCL, NOS; three EBV-positive DLBCL), one PBL, and four MCCHL.

### Diffuse Large B Cell Lymphoma, Not Otherwise Specified (DLBCL, NOS)

DLBCL, NOS developed in patients 04, 05, 19, and 21. Three patients presented with predominantly nodal DLBCL. Patient 04 had widespread nodal and extranodal involvement secondary to a EBV-associated lesion composed of polymorphous B cells responding to rituximab, received for recurrent autoimmune hemolytic anemia. Patient 05 had a preceding history of polymorphic, polyclonal EBV-associated LPD in the liver and nodal MZL both resolving after rituximab therapy. The subsequent EBV-negative DLBCL expressed MNDA (myeloid cell nuclear differentiation antigen) in a subpopulation suggesting a derivation from the nodal MZL. Panel sequencing of the lymphoma showed mutations in MYD88 (p.Ser219Cys) and NOTCH2 (p.Gln2409Ter). Patient 21 had been treated for nodular lymphocyte-predominant Hodgkin lymphoma (NLPHL) 12 years earlier. Subsequent lymph node biopsies were consistent with complete remission of NLPHL and finally nodal DLBCL. No mutations were identified by lymphoma panel sequencing. Patient 19 presented with extranodal DLBCL involving the duodenum. EBER was not detected in patients 19 and 21, and only present in rare small intratumoral bystander lymphocytes in patient 05. It could not be tested in patient 04. According to the immunohistochemical cell of origin classification (COO) [16, 28], two DLBCL, NOS cases were of the germinal center B cell (GCB) category (CD10 + IRF4/MUM1−; patient 20, 22) and two cases had a non-GCB phenotype (CD10-IRF4/MUM1+; patient 04, 05; Table 2).

PDL1 expression in tumor cells was detected in one (patient 05) out of three DLBCL, NOS cases by immunohistochemistry (Supplemental Table 1).

### EBV-Positive Diffuse Large B Cell Lymphoma

**Fig. 4:**
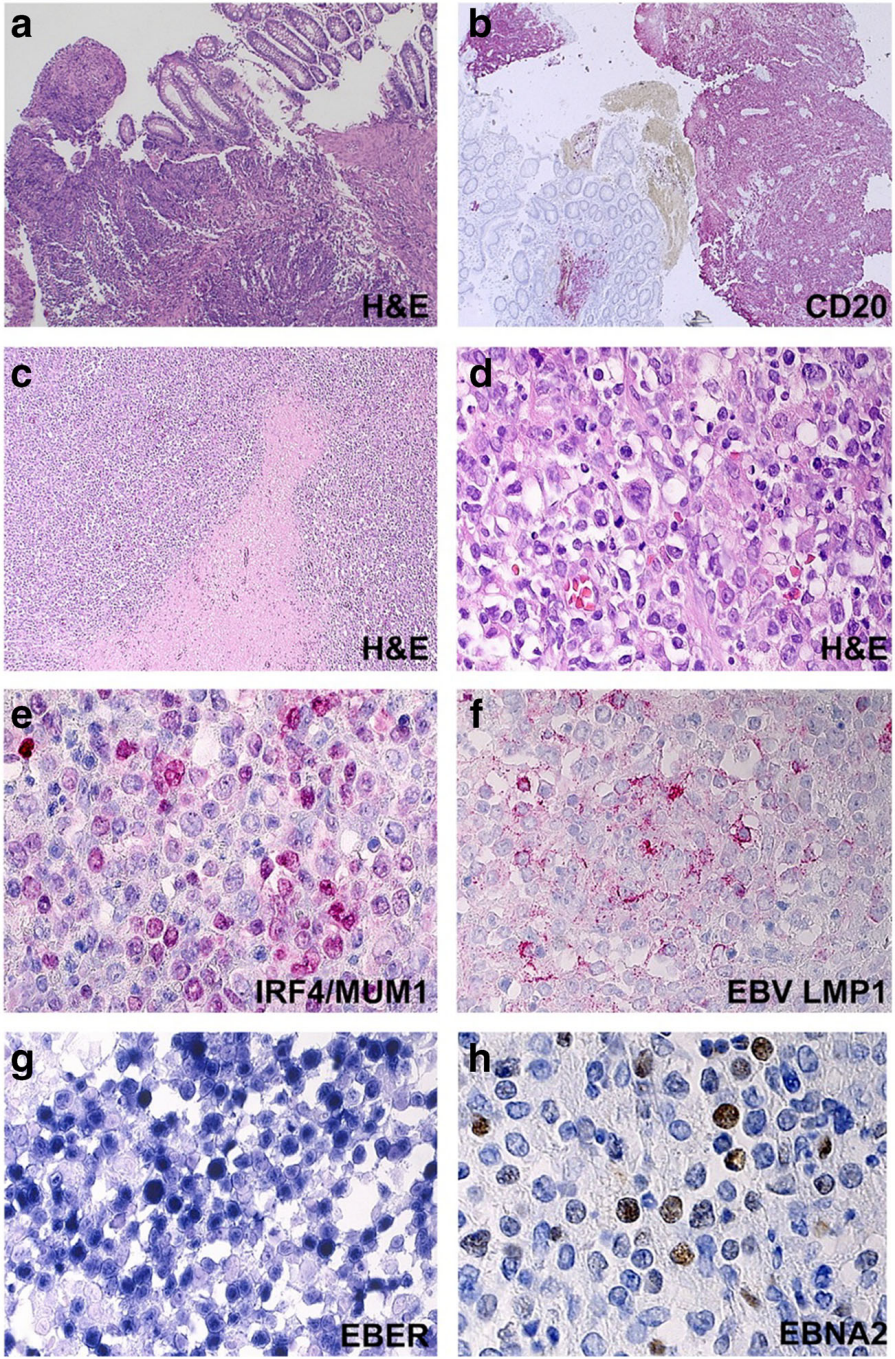
*EBV-positive diffuse large B-cell lymphoma (DLBCL).* Involvement of the large bowel (**a**, **b**) and spleen (**c**–**h**) (patient 1). Destruction of the intestinal mucosa and the adjacent large bowl wall by a large-sized CD20-positive B-cell population (**b**). Diffuse infiltration of the spleen by partially necrotic polymorphic blast cells positive for IRF4/MUM1 (**e**), EBV LMP1 (**f**), EBER (**g**), and EBNA2 (**h**)

This category includes three DLBCL cases in which EBER was detected in ≥80% of tumor cells (patient 01, 02, 09, Table 2, Supplemental Table 1). Patient 01 presented with extranodal DLBCL with multifocal involvement of the large bowel wall and the spleen (Fig. 4a–d), harboring a *BCL6* translocation. The EBV latency type III (EBER+, EBNA2+, LMP1+) was similar to post-transplant lymphoproliferative disorders (Fig. 4e–h). Patient 02 had a predominant DLBCL manifestation in the massively enlarged spleen and MNDA expression in the lymphoma supported a derivation from splenic marginal zone cells [29]. A common histologic feature of both cases was the polymorphism of tumor cells including bizarre giant cells and areas of necrosis. The nodal LPD of patient 09 showed a T cell/histiocyte-rich background. All three EBV-positive DLBCL cases belonged to the non-GCB subcategory. Two cases in this category (patients 01, 02) were tested for PDL1 and PD1 expression. Both showed a strong positivity for PDL1 by most tumor cells and TICs (histiocytes/dendritic cells) as well as for PD1 in tumor-infiltrating lymphocytes (Fig. 6c–d, Supplemental Table 1).

### Plasmablastic Lymphoma

Patient 13 was diagnosed with extranodal EBV+ PBL predominantly involving the rectal wall (Supplemental Fig. 1). The PBL showed a high proliferation fraction of 95%. Immunohistochemical staining suggested an EBV-latency type 1. EBV+ PBL has been reported to often express both PDL1 and PD1 [30]. In patient 13, the neoplastic population was PDL1 negative. The microenvironment contained rare PDL1+ TICs predominantly histiocytes/dendritic cells adjacent to tumor cells, but no PD1-positive cells (Fig. 6e, Supplemental Table 1).

### Classic Hodgkin Lymphoma

**Fig. 5:**
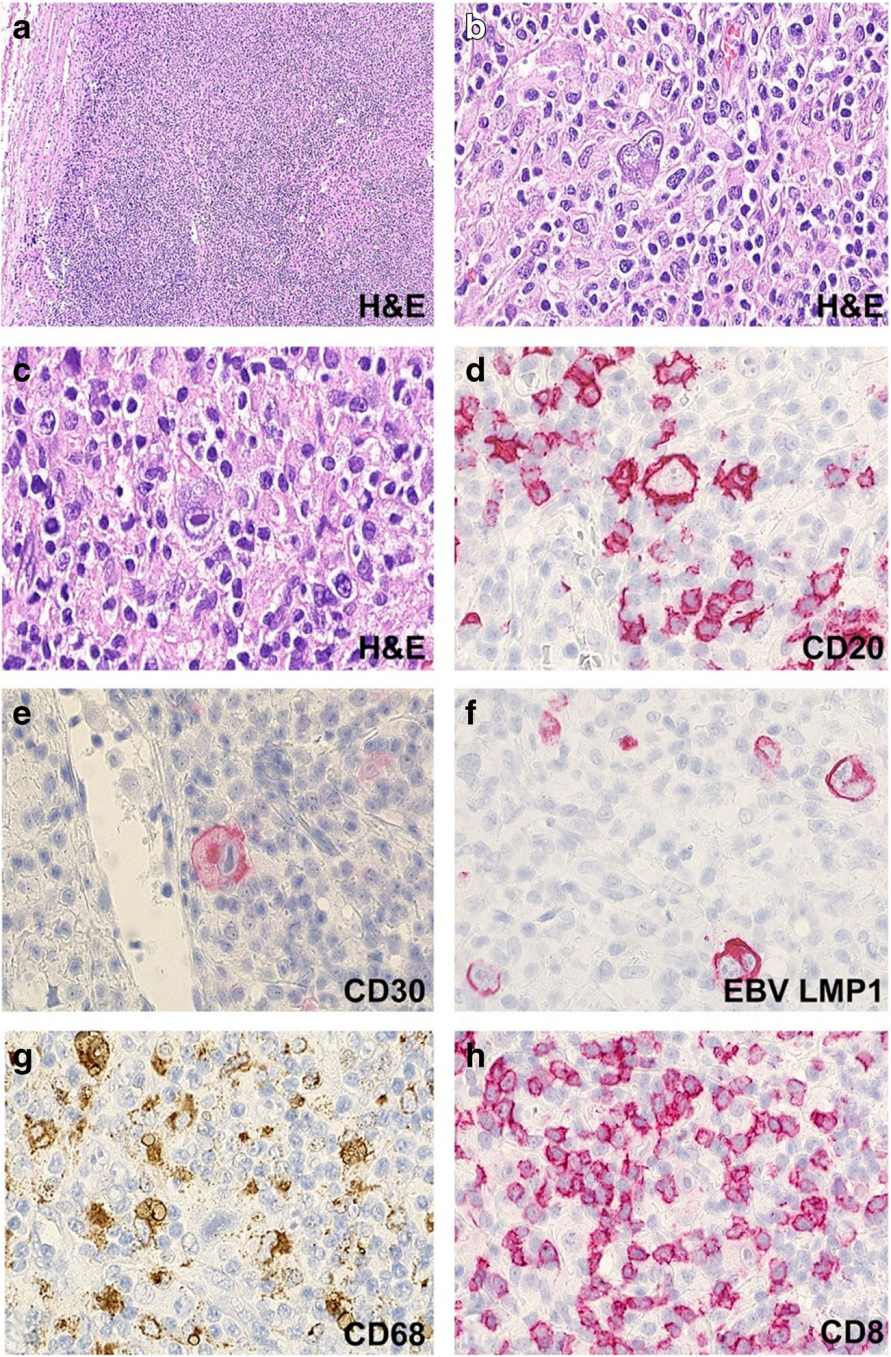
*Mixed cellularity classical Hodgkin lymphoma*. The lymph node structure (patient 06) is completely effaced and exhibits scattered typical Hodgkin- and Sternberg-Reed cells (**a**–**c**) expressing CD20 (**d**), CD30 (**e**), and EBV LMP1 (**f**). Reactive cells including CD68-positive histiocytes (G) and abundant CD8-positive T-cells represent the large majority of cells (H)

**Fig. 6:**
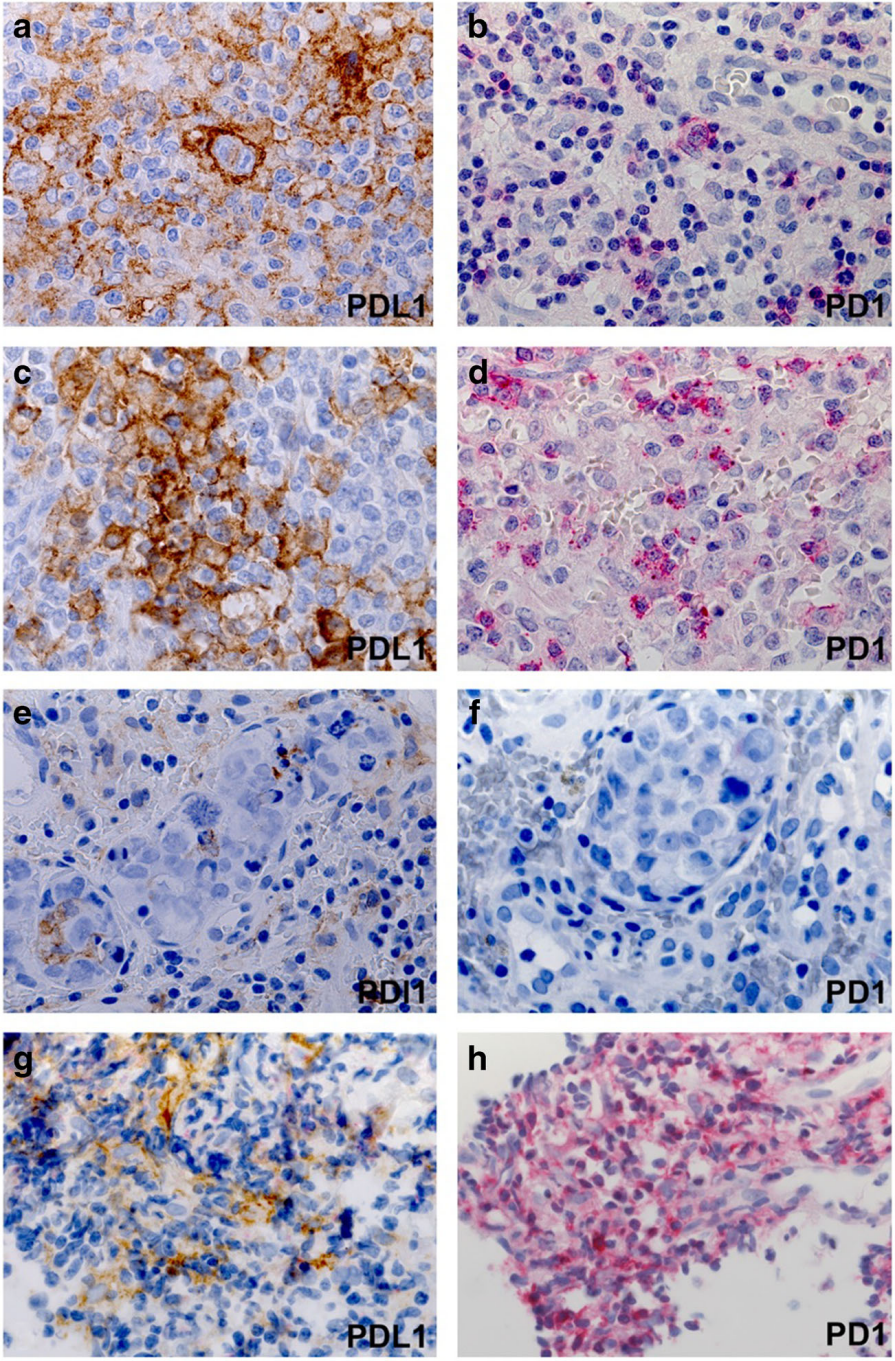
*PDL1 and PD1 expression in different lymphoma entities of the CVID cohort* (**a**–**b**) Mixed cellularity classical Hodgkin lymphoma (patient 06): PDL1 is strongly expressed in the neoplastic cells and the adjacent histiocytes and dendritic cells (**a**). The lymphoma also contains numerous PD1-positive small lymphocytes (**b**). (**c**–**d**): EBV-positive diffuse large B-cell lymphoma (patient 01): strong expression of PDL1 by large tumor cells (**c**) and of PD1 by reactive small lymphocytes (**d**). (**e**–**f**): Plasmablastic lymphoma (patient 13): PDL1 expression is restricted to reactive histiocytes and macrophages adjacent to completely negative tumor cells. Complete lack of PD1 immunoreactivity in the microenvironment (**f**). (**g**–**h**): Extranodal marginal zone lymphoma (patient 08): moderate expression of PDL1 by histiocytes and dendritic cells in extranodal MZL while lymphoma cells are negative (**g**). Abundant PD1-positive T-cells in the microenvironment of the same MZL specimen (**h**)

MCCHL developed in four patients (patients 06, 12, 18, 20, Fig. 5). Germline *CTLA4* mutations were detected in all tested patients (*n* = 3). The CHLs were assigned to EBV latency type II, which is characteristic for EBV + -CHL. EBV positivity was not restricted to the neoplastic population but also present in scattered small bystander cells. The microenvironment was abundantly composed of small T cells and large clusters of TICs, especially CD68+ histiocytes and protein S100 + CD11c + dendritic cells. The predominant T cell population expressed CD8 and cytotoxic molecules including granzyme B, perforin, and TIA. PDL1 was strongly expressed by tumor cells and TICs accompanied by abundant tumor-infiltrating PD1+ lymphocytes (Fig. 6a, Supplemental Table 1). Both PDL1- and PD1-positive cells were increased especially in the peripheral tumor tissue adjoining the surrounding non-neoplastic tissue.

### T Cell Lymphomas

Three CVID patients were diagnosed with T cell neoplasms including two T-LGLL cases and one PTCL, NOS.

### T Cell Large Granular Lymphocytic Leukemia (T-LGLL)

CD8+CD57+ T-LGLL developed in patients 10 and 11. T-LGLL did not only involve the peripheral blood and bone marrow but also infiltrated liver and colon (patient 10). Diagnosis was based on expansion of lymphocytes with typical LGL morphology and clonal rearrangement of the TCRɣ chain. Bone marrow infiltration by LGL cells was associated with reticulin fibrosis grade two and maturation defects of the three hematopoietic lineages (Supplemental Fig. 2) resulting in transfusion dependency. EBV testing of histologic specimens could not be performed.

### Peripheral T Cell Lymphoma, Not Otherwise Specified (PTCL, NOS)

Patient 14 had extensive nodal involvement by an EBV-negative PTCL, NOS at the age of 80 years after a lifelong course of an “infection only” CVID complicated by one single episode of ITP. Considering the rarity of T cell lymphomas described in CVID and the advanced age of the patient at diagnosis of the PTCL, the PTCL might not be a complication of the immunodeficiency but rather a co-incidence of two different diseases.

## Clinical Outcome in CVID Patients with Lymphoid Neoplasm

All patients with indolent B cell lymphoma (*n* = 6) and MCCHL (*n* = 4) were treated with immuno-chemotherapy or rituximab alone, and were alive at data retrieval. Patient 03 underwent allogeneic hematopoietic cell transplantation (alloHCT). The median duration of follow-up for indolent B cell lymphoma was 7.5 years (IQR 2.4–11.3) and for MCCHL 5 years (IQR 2.1–6). Of the patients with aggressive B cell lymphomas, four of eight patients were alive and in remission at data retrieval (median: 3.8 years, total range 0.7–14.7). Two of these patients underwent alloHCT. Four patients with aggressive B cell lymphomas died from either progression (*n* = 3) or neutropenic fever (*n* = 1); median time until death was 0.5 years (total range 0.1–2.1). One patient with T-LGLL died from progressive disease 5 years after diagnosis, and another was cured by alloHCT and in complete remission for >10 years at data retrieval. In summary, with the restrictions of a retrospective chart review and the limited number of patients, we conclude that the prognosis was predominantly determined by the underlying lymphoid neoplasm, while treatment toxicity and infections had a minor impact.

## Discussion

To our knowledge, this study summarizes the largest analysis of lymphoid neoplasms categorized according to the updated 2016 WHO classification [16] in patients diagnosed with CVID. Lymphoid malignancies developed at a younger median age in our cohort (38 years, IQR: 32–51) compared to the median age at lymphoma diagnosis in the general UK population (67.2 years, IQR: 54.9–76.5) [24]. The median latency of 14 years between the onset of CVID and diagnosis of lymphoma may not reflect the true latency since we had excluded lymphoma diagnosed before or within the first year of CVID diagnosis. The histopathologic review identified DLBCL (33%), MZL (29%), and MCCHL (19%) as the three most prevalent lymphoma subtypes. Among the MZL, five cases were ENMZL of MALT (*n* = 5, 24%) and one splenic MZL (*n* = 1, 5%). In the general UK population, DLBCL represented 40.9%, CHL 12.7%, and all MZL subtypes 17%. Importantly, ENMZL represented only 3.6% of lymphoid neoplasms in the general population [24] highlighting that MZL, with the caveat of statistics based on small numbers, is highly overrepresented in our and previous series [4,5]. From the literature, we retrieved 53 B cell lymphomas classified according to the current nomenclature (Supplemental Table 2). Among these, MZL represented 32% and HL 30% of cases comparable to 33% and 22% of B cell lymphomas in our cohort. The prevalence of DLBCLs (39%) was higher in our cohort compared to the literature (21%). Noteworthy, many B cell malignancies diagnosed in CVID, such as DLBCL, HL, and MZL, result from the malignant transformation of mature B cells that have experienced the germinal center reaction and usually carry somatic mutations of immunoglobulin genes but not from unmutated B cell populations. To our knowledge, mantle cell lymphomas that predominantly derive from mature B cells that do not enter the follicular germinal center and carry no or a limited number of *IGHV* somatic mutations are not reported among the CVID-associated lymphoid neoplasms. Follicular lymphoma, in which hypermutation and class switching occur early in the lymphoma development, is uncommon in CVID (3 of 53 cases [11, 13, 31, 32]). It was also not present in our cohort despite its prevalence of 16% in the general population [24].

Mechanisms increasing the risk for lymphoid neoplasms in immunodeficiency include chronic inflammation and immune stimulation, transforming viral events, decreased immunological tumor surveillance, and other host factors including genetics [33, 34]. These risk factors might differ between lymphoma subtypes. In line with this hypothesis, we found a high incidence of non-infectious, inflammatory complications and non-malignant LPD preceding the malignancy. In addition to the previously reported increased risk of developing lymphoma in patients with splenomegaly, lymphadenopathy [2, 12], and autoimmune cytopenia [13], we also found a higher prevalence of gastrointestinal complications in CVID lymphoma patients compared to the general CVID population. Inflammation caused by chronic antigenic stimulation, due to recurrent infections or autoimmune diseases at mucosal sites, induces the development of organized lymphoid tissue in EN sites. Clonal expansion of MZ cells leads to the development of MZL of MALT [35, 36], which represents the second most frequent B cell lymphoma subtype in our cohort. In three of four CVID patients, the primary site of MZL involvement was the lung, despite the association with chronic gastrointestinal inflammation. In fact, in the general population, the gastrointestinal tract is most often affected [37].

DLBCL, including DLBCL, NOS and EBV + -DLBCL, was the most frequent lymphoma entity in our cohort, comprising 39% of B cell lymphomas. Among the four DLBCL, NOS, one patient had a preceding history of “lymphadenitis” and two were preceded by either nodal MZL or NLPHL. Transformation of NLPHL into DLBCL occurs in 3–5% of cases even after a long latency period [16]. A potential transformation of MZL into DLBCL as part of the newly characterized C1 cluster of DLBCL [38] may underlie the lymphoma in patient 05, as it carried a mutation of *NOTCH2* that belongs to the frequently affected genes in splenic and nodal MZL. This hypothesis is also supported by expression of MNDA protein, however due to lack of material could not be molecularly confirmed. In addition, the DLBCL of patient 05 had a *MYD88*^*non-L265P*^ mutation that was enriched in the molecularly defined C1 cluster of DLBCL [38]. This cluster frequently harbors *NOTCH2* mutations, predominantly includes ABC-type tumors, and exhibits multiple bases of immune escape [38]. According to the COO designation for DLBCL, only two of seven cases in our cohort were of the GCB subgroup, while the remainder belonged to the non-GCB subgroup. These included the three EBV-associated DLBCLs. One GCB-type DLBCL contained no mutation by NGS analysis. No reliable data highlighting the genetic landscape and molecular pathogenesis of DLBCL in the context of CVID is currently available.

The link between EBV and tumor development in immunocompromised patients is well established [39]. We detected an EBV association in eight of 16 cases including DLBCL, PBL, and MCCHL. However, other classical EBV-associated malignancies (nasopharyngeal carcinoma, uncommon B cell lymphoma entities, T/NK-LPD/lymphomas, Burkitt lymphoma) were not present. The proportion of EBV-associated DLBCL was higher (43%) in our cohort compared to the general Western population (<5% of cases) [16, 40]. EBV+-DLBCLs are classified as a separate entity, often of an ABC subtype and express PDL1 on their surface. These lymphomas are more frequently associated with decreased tumor surveillance in immunodeficiency [16, 41–43]. Accordingly, the EBV+ DLBCLs in our cohort were of non-GCB type and showed PDL1 expression in tumor cells, supporting a tolerogenic environment. The four cases of CHL, all being of MCCHL subtype, were EBV positive and showed a tolerogenic environment with PDL1 and PD1 expression, especially in the peripheral areas of the tumor tissue. MCCHL is typically associated with human immunodeficiency virus infection [44] and is characterized by a dense infiltrate of non-malignant immune cells including CD4+, CD8+, and regulatory T cells [45]. Remarkably, three of four patients with MCCHL carried germline *CTLA4* mutations. The EBV-association rate for CHL in the general population of Western countries is about 30–35%, but for MCCHL, it is as high as 75% [16, 39]. The genetic background of *CTLA4* mutations may play an important role in the surveillance of EBV-driven transformation and the predisposition to MCCHL. Reduced CTLA4 expression on tumor-infiltrating T cells might permit increased expression of CD80/CD86 on Hodgkin-Reed-Sternberg cells, thereby increasing T cell proliferation and modifying the tumor environment.

Lymphomas of T cell lineage are reported in a small number of CVID patients. Riaz et al. [11] included nine cases and the Czech nation-wide study two cases [13]. We diagnosed a T-LGLL in two patients and a PTCL, NOS in an additional patient. In CVID patients, increased numbers of polyclonal LGLs associated with neutropenia have been reported [21], and in one case, granulomatous lymphocytic interstitial lung disease was detected [22]. Potentially triggered by a chronic persistent stimulus, T-LGLL carries somatic *STAT3* mutations in about 30–40% of cases and is often associated with autoimmune disorders [16, 46]. Germline gain-of-function mutations in *STAT3* have been identified in some CVID-like patients [47]. The *STAT3* mutational status of our T-LGLL patients is unknown; however, both had preceding lymphoid hyperplasia and autoimmune phenomena supporting the concept of an underlying inflammatory condition.

While it is attractive to speculate that the reduction of the discussed predisposing risk factors is a reasonable approach to reduce the lymphoma burden in CVID, there is insufficient data to conclude if and how non-malignant LPD and chronic inflammation can be successfully treated in the context of CVID and if this will reduce the risk of a lymphoid malignancy. On the other hand, immunosuppression might even elevate the risk of lymphoma development as it has been shown for the use of thiopurines and/or anti-TNF treatment in inflammatory bowel disease [48].

Currently, the treatment of lymphoid neoplasms is following treatment guidelines for immunocompetent patients [33]. Specific findings in immunodeficiency-associated lymphomas may be relevant weighing the different therapeutic options. The high percentage of EBV association and evidence of a tolerogenic tumor environment in CVID-associated DLBCL and CHL suggest that they may be candidates for immune checkpoint inhibitor therapy. The potential risks and benefits of checkpoint inhibitor therapy in patients with immune dysregulation have to be evaluated individually. In general, prognosis and treatment of B/T cell lymphomas depend on subtype, risk factors, and tumor stage at diagnosis. Meaningful tumor staging is often difficult in CVID patients due to preceding lymphoid hyperplasia, raising concern for comparisons to the general population’s outcome data. Nonetheless, even when considering stage IV disease for all CVID patients, a mortality rate of 50% in DLBCL patients after 6 months is high compared to the general population and the German HIV-related lymphoma cohort [49]. Thus, given the early onset of lymphoid neoplasms in CVID, the dismal prognosis, and the genetic predisposition in some of the patients, we suggest to consider alloHCT in first remission [50, 51].

Overall, our data supports that autoimmune manifestations and non-malignant LPD are risk factors for the development of lymphoid malignancies in CVID. The spectrum of lymphomas differs in several aspects from the general population and most likely reflects the underlying immunodeficiency as a pathogenetically relevant factor. In the majority of patients, the exact risk factors and mechanisms initiating tumorigenesis and promoting progression to overt lymphoma still remain elusive and may be linked to different molecular subgroups. In the future, international collaborative efforts will have to shed light into these mechanisms of lymphomagenesis in CVID. Multicenter trial designs should include clinically and genetically defined patients, a central review of non-malignant and malignant LPD and the molecular landscape of tumor samples and preceding non-malignant lesions.

## Supplementary Information

ESM 1 (DOCX 22 kb)

ESM 2 (PDF 19 kb)

ESM 3 (DOCX 17 kb)

ESM 4 (PDF 5786 kb)

ESM 5 (PDF 6894 kb)

## Abbreviations

alloHCT: Allogeneic hematopoietic cell transplantation
CHL: Classic Hodgkin lymphoma
CVID: Common variable immunodeficiency
DLBCL: Diffuse large B cell lymphoma
EBV: Epstein-Barr virus
ENMZL: Extranodal marginal zone lymphoma
loCID: Late-onset combined immunodeficiency
LPD: Lymphoproliferative disorders
MALT: Mucosa-associated lymphoid tissue
MCCHL: Mixed-cellularity classic Hodgkin lymphoma
MNDA: Myeloid cell nuclear differentiation antigen
MZL: Marginal zone lymphoma
NLPHL: Nodular lymphocyte-predominant Hodgkin lymphoma
PBL: Plasmablastic lymphoma
PTCL, NOS: Peripheral T cell lymphoma, not otherwise specified
SMZL: Splenic marginal zone lymphoma
T-LGLL: T cell large granular lymphocytic leukemia
TICs: Tumor-infiltrating cells
WES: Whole-exome sequencing
