# Supplementary material for: Altered Spectrum of Lymphoid Neoplasms in a Single-Center Cohort of Common Variable Immunodeficiency with Immune Dysregulation
Source: J Clin Immunol. 2021 Apr 19;41(6):1250–65. doi: 10.1007/s10875-021-01016-4 (PMC8310845; doi:10.1007/s10875-021-01016-4)
Supplement: Supplementary file 1 — (DOCX 22 kb) [file 10875_2021_1016_MOESM1_ESM.docx]

# Supplemental Methods

## Patient cohort and data collection

Patient characteristics are summarized in Supplemental Table 1. All patients signed informed consent. Late onset combined immunodeficiency (loCID) was defined as previously published[^1^](https://paperpile.com/c/Xp7f4M/dJsvv). Patients 01, 05, 09, 11 were reported previously^[2](https://paperpile.com/c/Xp7f4M/mh1Xl)^. Splenomegaly was defined as sonographic spleen size >4.7x11cm. Inflammatory lung involvement was defined as either granulomatous or lymphocytic interstitial lung disease excluding bronchiectasis. Gastrointestinal involvement included nodular lymphoid hyperplasia, seronegative enteropathy similar to celiac disease with villous atrophy and intraepithelial lymphocytosis, microscopic enteritis and/or colitis with minimal mucosal alterations, or Crohn-like disease. Unexplained diarrhea and wasting in patients with negative or unavailable biopsies and negative stool cultures for enteropathogenic microbiota were also considered as gastrointestinal manifestation of CVID. Hepatobiliary complications included polyclonal LPD, autoimmune cholangitis or nodular regenerative hyperplasia.

## Targeted sequencing of germline DNA

DNA was extracted from peripheral blood mononuclear cells or granulocytes. Whole exome sequencing (WES) was performed. Results were screened for mutations in combined immunodeficiency-, antibody deficiency-, or immune dysregulation-related genes according to tables 1, 3 and 4 in[^3^](https://paperpile.com/c/Xp7f4M/OoAWt). The raw sequenced reads stored in FASTQ files were mapped against the human reference genome build UCSC hg19 using Bowtie 2 v2.2.3[^4^](https://paperpile.com/c/Xp7f4M/Vd2cd), reordered, sorted and converted to BAM format, followed by the removal of PCR duplicates with Picard v1.115 (<http://picard.sourceforge.net>). Local realignment around InDels and base quality score recalibration as well as variant calling and variant quality score recalibration were performed with the GATK v3.1[^5^](https://paperpile.com/c/Xp7f4M/3HgtM) according to their best practice recommendations. Genetic variation data stored in VCF format files was handled using VCFtools program package[^6^](https://paperpile.com/c/Xp7f4M/ebbrj). For the annotation of discovered variants with the IDs from the short genetic variations database dbSNP v142 (<http://www.ncbi.nlm.nih.gov/SNP/>) the program SnpSift was used, which is part of the main distribution of the toolbox SnpEff v3.6 (<http://snpeff.sourceforge.net>) by[^7^](https://paperpile.com/c/Xp7f4M/KVQ0O). The annotation of variants with the genes and transcripts they are affecting and the effects they produce was conducted using the effect prediction tool SnpEff. Patient 20 underwent targeted sequencing of the CTLA4 locus.

## Histologic evaluation of tissue specimens

### Histology and immunohistochemistry

Archival formalin-fixed paraffin-embedded (FFPE) specimens obtained at the initial diagnosis of a lymphoid neoplasm covered the years 2001-2018. Previous biopsies from gut, lymph nodes or bone marrow were reviewed, if available. Routine conventional stains (hematoxylin and eosin, giemsa, periodic-acid Schiff) were complemented by immunohistochemistry using commercially available antibodies for CD3, CD5, CD10, CD15, CD20, CD21, CD23, CD30, CD68, BCL2, BCL6, cyclin D1, IRF4/MUM1, EBV-latent membrane protein (LMP-1), Ki67, kappa and lambda. Additional stains performed at reevaluation included CD4, CD8, PRDM1/Blimp-1, EBV nuclear antigen 2 (EBNA2), granzyme B, perforin, TIA, IgA, IgD, IgG, IgM, MYC, protein S100 and polyclonal rat anti-MNDA (myeloid cell nuclear differentiation antigen) antibody. The sections were evaluated and documented using a Zeiss Imager.M1 with an adapted imaging camera (Carl Zeiss, Oberkochen, Germany).

Since only FFPE material was available, the cell of origin classification (COO) based on the expression of CD10, BCL6 and IRF4/MUM1 was used as surrogate marker for the germinal center B-cell-like (GCB) vs. the non-GCB immunohistochemical subgroups of DLBCL[^8,9^](https://paperpile.com/c/Xp7f4M/57ogG+utxr).

EBV status was evaluated by immunohistochemistry and chromogen in-situ hybridisation (CISH) against EBV encoded small RNA (EBER, ZytoVision, Bremerhaven, Germany).

In B-cell lymphoproliferations fluorescence-in situ hybridization (FISH) was performed using MYC, BCL2 and BCL6 dual color break apart rearrangement probes (Abbott Molecular Inc, Des Plaines, IL 60018, USA).

Polymerase chain reaction (PCR) studies to detect immunoglobulin heavy chain (IgH)- and T-cell receptor rearrangement using BIOMED-2 primer sets were performed on DNA extracted from FFPE tissue blocks[^10^](https://paperpile.com/c/Xp7f4M/thIr5).

FFPE material for immunohistochemical assessment of PD-L1 (PDL-1, rabbit, Roche-Ventana, Sp263) and PD1 (goat, R&D System AF1086) was available in 11/21 cases. PD-L1 and PD1 staining was evaluated in tumor cells (TC) and in non-neoplastic tumor infiltrating cells (TIC) especially in lymphocytes, histiocytes/dendritic cells of the microenvironment. A semiquantitative scoring was performed by assessing the percentage of positive cells: score 0 = <1%, 1 = 1-5%, score 2 = 6-50%, score 3 = 51-100%.

### Targeted sequencing of lymphoid neoplasms

The Illumina TruSight Lymphoid Panel was used for targeted sequencing of DNA extracted from FFPE lymphoma samples (QIAamp DNA FFPE Tissue Kit, Qiagen, Hilden, Germany) and processed as described by the manufacturer (Illumina Inc., San Diego, CA). Libraries were sequenced on an Illumina NextSeq 550 and the resulting bcl2 files converted to FASTQ with the Illumina Local run Manager software and the bcltofastq module. FASTQ-files were further analysed with the SeqNext software (JSI Medical Systems, Ettenheim, Germany). We used a significance threshold of 5% for the detection of variants, with a minimum coverage of 2000 reads and minimum 200 reads per variant in both directions. We set the Q-Score threshold to 20 and considered only reads where more than 40% of bases reached this threshold. Variants with entries in SNP databases that had variant allele frequencies around 50%, with minor allele frequencies greater 1% in the general population and that were no proven somatic mutations were considered germline variants and excluded.

# Supplemental Results

## Characteristics of the patient cohort: germline genetic analysis

In our cohort of 21 adult patients with a diagnosis of CVID and lymphoid neoplasm, six patients fulfilled the sub-criteria of late onset combined immunodeficiency (loCID). Of 13 patients analysed, disease associated variants or mutations were identified in five cases (Supplemental Table 1): three patients had previously published mutations in cytotoxic T-lymphocyte associated protein 4 (*CTLA4)*[*^11,12^*](https://paperpile.com/c/Xp7f4M/H3CRX+gqax7) and were also reported by Egg et al.[^13^](https://paperpile.com/c/Xp7f4M/Q5IYH). Patient 01 had a published *BACH2* mutation^[14](https://paperpile.com/c/Xp7f4M/1nT7u)^. Patient 07 carried a mutation in *TNFRSF13B*[*^15^*](https://paperpile.com/c/Xp7f4M/H0Omq), a known variant predisposing to CVID. Patient 15 showed a previously reported gain-of-function *STAT1* mutation[^16^](https://paperpile.com/c/Xp7f4M/ChtIT); she, however, exhibited normal STAT phosphorylation after IFNɑ or IFNɣ stimulation of monocytes and peripheral blood mononuclear cells (data not shown). Patient 09 carried a variant in *NFKB2* with unknown significance. Thus, disease associated variants were proven in 5/13 patients: three *CTLA4* one *BACH2* and one *TNFSR13B*.

# Supplemental References

1. [Malphettes M, Gérard L, Carmagnat M, et al. Late-onset combined immune deficiency: a subset of common variable immunodeficiency with severe T cell defect. Clin Infect Dis 2009;49(9):1329–1338.](http://paperpile.com/b/Xp7f4M/dJsvv)

2. [Wehr C, Gennery AR, Lindemans C, et al. Multicenter experience in hematopoietic stem cell transplantation for serious complications of common variable immunodeficiency. J Allergy Clin Immunol 2015;135(4):988–97.e6.](http://paperpile.com/b/Xp7f4M/mh1Xl)

3. [Picard C, Bobby Gaspar H, Al-Herz W, et al. International Union of Immunological Societies: 2017 Primary Immunodeficiency Diseases Committee Report on Inborn Errors of Immunity. J Clin Immunol 2018;38(1):96–128.](http://paperpile.com/b/Xp7f4M/OoAWt)

4. [Langmead B, Salzberg SL. Fast gapped-read alignment with Bowtie 2. Nat Methods 2012;9(4):357–359.](http://paperpile.com/b/Xp7f4M/Vd2cd)

5. [McKenna A, Hanna M, Banks E, et al. The Genome Analysis Toolkit: a MapReduce framework for analyzing next-generation DNA sequencing data. Genome Res 2010;20(9):1297–1303.](http://paperpile.com/b/Xp7f4M/3HgtM)

6. [Danecek P, Auton A, Abecasis G, et al. The variant call format and VCFtools. Bioinformatics 2011;27(15):2156–2158.](http://paperpile.com/b/Xp7f4M/ebbrj)

7. [Cingolani P, Platts A, Wang LL, et al. A program for annotating and predicting the effects of single nucleotide polymorphisms, SnpEff: SNPs in the genome of Drosophila melanogaster strain w1118; iso-2; iso-3. Fly 2012;6(2):80–92.](http://paperpile.com/b/Xp7f4M/KVQ0O)

8. [Hans CP, Weisenburger DD, Greiner TC, et al. Confirmation of the molecular classification of diffuse large B-cell lymphoma by immunohistochemistry using a tissue microarray. Blood 2004;103(1):275–282.](http://paperpile.com/b/Xp7f4M/57ogG)

9. [Swerdlow SH, Campo E, Harris NL, et al., editors. WHO Classification of Tumours of Haematopoietic and Lymphoid Tissues. 4th ed. International Agency for Research on Cancer; 2017.](http://paperpile.com/b/Xp7f4M/utxr)

10. [van Krieken JHJM, Langerak AW, Macintyre EA, et al. Improved reliability of lymphoma diagnostics via PCR-based clonality testing: report of the BIOMED-2 Concerted Action BHM4-CT98-3936. Leukemia 2007;21(2):201–206.](http://paperpile.com/b/Xp7f4M/thIr5)

11. [Schwab C, Gabrysch A, Olbrich P, et al. Phenotype, penetrance, and treatment of 133 cytotoxic T-lymphocyte antigen 4-insufficient subjects. J Allergy Clin Immunol [Epub ahead of print].](http://paperpile.com/b/Xp7f4M/H3CRX)

12. [Schubert D, Bode C, Kenefeck R, et al. Autosomal dominant immune dysregulation syndrome in humans with CTLA4 mutations. Nat Med 2014;20(12):1410–1416.](http://paperpile.com/b/Xp7f4M/gqax7)

13. [Egg D, Schwab C, Gabrysch A, et al. Increased Risk for Malignancies in 131 Affected CTLA4 Mutation Carriers. Front Immunol 2018;92012.](http://paperpile.com/b/Xp7f4M/Q5IYH)

14. [Afzali B, Grönholm J, Vandrovcova J, et al. BACH2 immunodeficiency illustrates an association between super-enhancers and haploinsufficiency. Nat Immunol 2017;18(7):813–823.](http://paperpile.com/b/Xp7f4M/1nT7u)

15. [Salzer U, Chapel HM, Webster ADB, et al. Mutations in TNFRSF13B encoding TACI are associated with common variable immunodeficiency in humans. Nat Genet 2005;37(8):820–828.](http://paperpile.com/b/Xp7f4M/H0Omq)

16. [Uzel G, Sampaio EP, Lawrence MG, et al. Dominant gain-of-function STAT1 mutations in FOXP3 wild-type immune dysregulation-polyendocrinopathy-enteropathy-X-linked-like syndrome. J Allergy Clin Immunol 2013;131(6):1611–1623.](http://paperpile.com/b/Xp7f4M/ChtIT)

#

# Supplemental Figure Legends

Supplemental Figure 1: *Plasmablastic lymphoma*

Cohesive infiltrate of large-sized blasts with eccentric nuclei and a basophilic cytoplasm in the rectal wall (patient 13). The lymphoma cells are negative for CD20 (B) but express CD138 (C), show Kappa light chain restriction (D) and are positive IRF4/MUM1 (E) and PRMD1/BLIMP1 (F). Note positivity for EBER (G) and EBNA2 (H) in the large majority of tumor cells (EBV-LMP1 was negative) suggesting an EBV-latency type 1.

Supplemental Figure 2: *T-cell large granular lymphocytic leukemia (T-LGLL)*

(A-H): Age-related moderately hypercellular bone marrow core biopsy (patient 11) showing small hypolobated megakaryocytes mimicking myelodysplastic features (A, B). Interstitial and intrasinusoidal infiltration by large lymphocytes exhibiting cytoplasmatic granulation and irregular surface protrusions at high magnification (C, D). Strong surface CD3 expression by the lymphoid cells highlighting the typical infiltration pattern of the bone marrow by T-LGLL (G, H).
