# Supplementary material for: Altered Spectrum of Lymphoid Neoplasms in a Single-Center Cohort of Common Variable Immunodeficiency with Immune Dysregulation
Source: J Clin Immunol. 2021 Apr 19;41(6):1250–65. doi: 10.1007/s10875-021-01016-4 (PMC8310845; doi:10.1007/s10875-021-01016-4)
Supplement: Supplementary file 2 — (PDF 19 kb) [file 10875_2021_1016_MOESM2_ESM.pdf]

Supplemental Table 1: EBV and PD1/PDL-1 status in B-cell lymphomas.

Patient	Lymphoid neplasm	PDL1 score, TC	PDL1 score, *TIC	PD1 score, §TIC	EBV, TC	EBV, TIC (rare)
7	extranodal MZL	0	2	3	negative	positive
8	extranodal MZL	0	2	3	negative	negative
15	extranodal MZL	0	2	2	negative	negative
16	extranodal MZL	.	.	.	.	.
17	extranodal MZL	.	.	.	.	.
3	splenic MZL	0	2	3	negative	positive
4	DLBCL, NOS	.	.	.	negative	.
5	DLBCL, NOS	2	2	3	negative	positive
19	DLBCL, NOS	0	2	2	negative	negative
21	DLBCL, NOS	0	3	2	negative	negative
1	EBV+ DLBCL, NOS	3	2	3	positive	positive
2	EBV+ DLBCL, NOS	3	2	3	positive	positive
9	EBV+ DLBCL, NOS	.	.	.	positive	.
13	PBL	0	2	0	positive	positive
6	MCCHL	3	3	2	positive	positive
12	MCCHL	.	.	.	positive	.
18	MCCHL	.	.	.	positive	.
20	MCCHL	3	3	3	positive	positive

Abbreviations: DLBCL: diffuse large B-cell lymphoma, MCCHL: Mixed cellularity classic Hodgkin lymphoma, MZL: marginal zone lymphoma, MALT: mucosa-associated lympoid tissue, NOS: not otherwise specified, PBL: plasmablastic lymphoma, TC: tumor cell, TIC: tumor infiltrating cells; *TIC: histiocytes/dendritic cells; §TIC: lymphocytes, T-LGLL: T-cell large granular lymphocytic leukemia, PTCL, NOS: Peripheral T cell lymphoma, not otherwise specified (PTCL, NOS). .: not evaluated

PDL1/PD1 score: A semiquantitative scoring was performed by assessing the percentage of positive cells: score 0 = <1%, 1 = 1-5%, score 2 = 6-50%, score 3 = 51-100%.
