# Supplementary material for: Altered Spectrum of Lymphoid Neoplasms in a Single-Center Cohort of Common Variable Immunodeficiency with Immune Dysregulation
Source: J Clin Immunol. 2021 Apr 19;41(6):1250–65. doi: 10.1007/s10875-021-01016-4 (PMC8310845; doi:10.1007/s10875-021-01016-4)
Supplement: Supplementary file 3 — (DOCX 17 kb) [file 10875_2021_1016_MOESM3_ESM.docx]

### Supplemental Table 2: B-cell lymphomas classified according to the current WHO classification occurring in the context of CVID

| **Reference** | **Number of patients** | **Distribution of lymphoma entities** |
| --- | --- | --- |
| [(Aghamohammadi et al. 2006)](https://paperpile.com/c/Dx5zLi/5ATO) | 1 | 1 ENMZL |
| [(Spickett 2001)](https://paperpile.com/c/Dx5zLi/SmrP) | 1 | 1 ENMZL |
| [(Rael et al. 2016)](https://paperpile.com/c/Dx5zLi/v0Iz) | 1 | 1 HL |
| [(Cunningham-Rundles and Bodian 1999)](https://paperpile.com/c/Dx5zLi/ZVi5) | 3 | 3 HL |
| [(Cunningham-Rundles et al. 2002)](https://paperpile.com/c/Dx5zLi/AHXD) | 5 | 5 ENMZL |
| [(Cunningham-Rundles et al. 1991)](https://paperpile.com/c/Dx5zLi/BP4m) and [(Cunningham-Rundles et al. 1987)](https://paperpile.com/c/Dx5zLi/AOOE) | 6 | 5 DLBCL  1 FL |
| [(Mellemkjaer et al. 2002)](https://paperpile.com/c/Dx5zLi/65ZO) | 1 | 1 HL |
| [(Castellano et al. 1992)](https://paperpile.com/c/Dx5zLi/ig74) | 1 | 1 MZL |
| [(Hermaszewski and Webster 1993)](https://paperpile.com/c/Dx5zLi/MccF) | 1 | 1 HL |
| [(Reichenberger et al. 2001)](https://paperpile.com/c/Dx5zLi/kF0H) | 1 | 1 ENMZL |
| [(Vajdic et al. 2010)](https://paperpile.com/c/Dx5zLi/sfgc) | 1 | 1 DLBCL |
| [(Resnick et al. 2012)](https://paperpile.com/c/Dx5zLi/7Wn6) | 20 | 6 ENMZL  1 MZL  4 HL  3 DLBCL  2 FL  1 BL  1 SLL  1 WM  1 T-cell rich B-cell lymphoma |
| [(Kralickova et al. 2018)](https://paperpile.com/c/Dx5zLi/Q5WF) | 9 | 5 HL  2 DLBCL  1 BL  1 MZL |
| [(Stenton et al. 2016)](https://paperpile.com/c/Dx5zLi/F8wE) | 1 | 1 DLBCL |
| [(Malkan et al. 2015)](https://paperpile.com/c/Dx5zLi/0DeV) | 1 | 1 HL |

Only published B-cell lymphomas classified according to the 2016 WHO classification are listed. The data are extracted from Table 1 in Riaz et al [(Riaz et al. 2019)](https://paperpile.com/c/Dx5zLi/i4Ik).

### References

[Aghamohammadi, Asghar, Nima Parvaneh, Farrokh Tirgari, Fatemeh Mahjoob, Masoud Movahedi, Mohammad Gharagozlou, Mahboubeh Mansouri, Ali Kouhi, Nima Rezaei, and David Webster. 2006. “Lymphoma of Mucosa-Associated Lymphoid Tissue in Common Variable Immunodeficiency.” *Leukemia & Lymphoma* 47 (2): 343–46.](http://paperpile.com/b/Dx5zLi/5ATO)

[Castellano, G., D. Moreno, O. Galvao, C. Ballestín, F. Colina, M. Mollejo, J. D. Morillas, and J. A. Solís Herruzo. 1992. “Malignant Lymphoma of Jejunum with Common Variable Hypogammaglobulinemia and Diffuse Nodular Hyperplasia of the Small Intestine. A Case Study and Literature Review.” *Journal of Clinical Gastroenterology* 15 (2): 128–35.](http://paperpile.com/b/Dx5zLi/ig74)

[Cunningham-Rundles, C., and C. Bodian. 1999. “Common Variable Immunodeficiency: Clinical and Immunological Features of 248 Patients.” *Clinical Immunology*  92 (1): 34–48.](http://paperpile.com/b/Dx5zLi/ZVi5)

[Cunningham-Rundles, C., Dennis L. Cooper, Thomas P. Duffy, and James Strauchen. 2002. “Lymphomas of Mucosal-Associated Lymphoid Tissue in Common Variable Immunodeficiency.” *American Journal of Hematology* 69 (3): 171–78.](http://paperpile.com/b/Dx5zLi/AHXD)

[Cunningham-Rundles, C., P. Lieberman, G. Hellman, and R. S. Chaganti. 1991. “Non-Hodgkin Lymphoma in Common Variable Immunodeficiency.” *American Journal of Hematology* 37 (2): 69–74.](http://paperpile.com/b/Dx5zLi/BP4m)

[Cunningham-Rundles, C., F. P. Siegal, S. Cunningham-Rundles, and P. Lieberman. 1987. “Incidence of Cancer in 98 Patients with Common Varied Immunodeficiency.” *Journal of Clinical Immunology* 7 (4): 294–99.](http://paperpile.com/b/Dx5zLi/AOOE)

[Hermaszewski, R. A., and A. D. Webster. 1993. “Primary Hypogammaglobulinaemia: A Survey of Clinical Manifestations and Complications.” *The Quarterly Journal of Medicine* 86 (1): 31–42.](http://paperpile.com/b/Dx5zLi/MccF)

[Kralickova, Pavlina, Tomas Milota, Jiri Litzman, Ivana Malkusova, Dalibor Jilek, Jitka Petanova, Jana Vydlakova, et al. 2018. “CVID-Associated Tumors: Czech Nationwide Study Focused on Epidemiology, Immunology, and Genetic Background in a Cohort of Patients With CVID.” *Frontiers in Immunology* 9: 3135.](http://paperpile.com/b/Dx5zLi/Q5WF)

[Malkan, Umit Yavuz, Gursel Gunes, Tuncay Aslan, Sezgin Etgul, Seda Aydin, and Yahya Buyukasik. 2015. “Common Variable Immune Deficiency Associated Hodgkin’s Lymphoma Complicated with EBV-Linked Hemophagocytic Lymphohistiocytosis: A Case Report.” *International Journal of Clinical and Experimental Medicine* 8 (8): 14203–6.](http://paperpile.com/b/Dx5zLi/0DeV)

[Mellemkjaer, L., L. Hammarstrom, V. Andersen, J. Yuen, C. Heilmann, T. Barington, J. Bjorkander, and J. H. Olsen. 2002. “Cancer Risk among Patients with IgA Deficiency or Common Variable Immunodeficiency and Their Relatives: A Combined Danish and Swedish Study.” *Clinical and Experimental Immunology* 130 (3): 495–500.](http://paperpile.com/b/Dx5zLi/65ZO)

[Rael, Efren, Kevin Rakszawski, Kristian Koller, Michael Bayerl, Manish Butte, and Hong Zheng. 2016. “Treatment with Rituximab and Brentuximab Vedotin in a Patient of Common Variable Immune Deficiency-Associated Classic Hodgkin Lymphoma.” *Biomarker Research* 4 (March): 7.](http://paperpile.com/b/Dx5zLi/v0Iz)

[Reichenberger, F., C. Wyser, M. Gonon, G. Cathomas, and M. Tamm. 2001. “Pulmonary Mucosa-Associated Lymphoid Tissue Lymphoma in a Patient with Common Variable Immunodeficiency Syndrome.” *Respiration; International Review of Thoracic Diseases* 68 (1): 109–12.](http://paperpile.com/b/Dx5zLi/kF0H)

[Resnick, Elena S., Erin L. Moshier, James H. Godbold, and Charlotte Cunningham-Rundles. 2012. “Morbidity and Mortality in Common Variable Immune Deficiency over 4 Decades.” *Blood* 119 (7): 1650–57.](http://paperpile.com/b/Dx5zLi/7Wn6)

[Riaz, Irbaz Bin, Warda Faridi, Mrinal M. Patnaik, and Roshini S. Abraham. 2019. “A Systematic Review on Predisposition to Lymphoid (B and T Cell) Neoplasias in Patients With Primary Immunodeficiencies and Immune Dysregulatory Disorders (Inborn Errors of Immunity).” *Frontiers in Immunology* 10 (April): 777.](http://paperpile.com/b/Dx5zLi/i4Ik)

[Spickett, G. P. 2001. “Current Perspectives on Common Variable Immunodeficiency (CVID).” *Clinical and Experimental Allergy: Journal of the British Society for Allergy and Clinical Immunology* 31 (4): 536–42.](http://paperpile.com/b/Dx5zLi/SmrP)

[Stenton, Sophie, Malee Fernando, Zanna Currie, and Hardeep Singh Mudhar. 2016. “Metachronous Diffuse Large B-Cell Lymphoma and Kaposi Sarcoma of the Right Eyelid and Lacrimal Gland in a Patient with Granulomatous Common Variable Immunodeficiency.” *Ocular Oncology and Pathology* 2 (3): 197–201.](http://paperpile.com/b/Dx5zLi/F8wE)

[Vajdic, Claire M., Limin Mao, Marina T. van Leeuwen, Philippa Kirkpatrick, Andrew E. Grulich, and Sean Riminton. 2010. “Are Antibody Deficiency Disorders Associated with a Narrower Range of Cancers than Other Forms of Immunodeficiency?” *Blood* 116 (8): 1228–34.](http://paperpile.com/b/Dx5zLi/sfgc)
