# Supplementary figures and images for: Altered Spectrum of Lymphoid Neoplasms in a Single-Center Cohort of Common Variable Immunodeficiency with Immune Dysregulation
Source: J Clin Immunol. 2021 Apr 19;41(6):1250–65. doi: 10.1007/s10875-021-01016-4 (PMC8310845; doi:10.1007/s10875-021-01016-4)

## Supplemental Figure 1

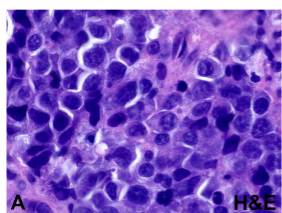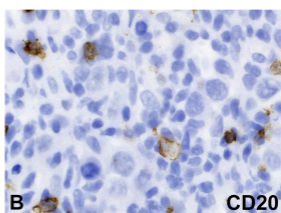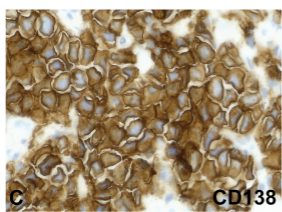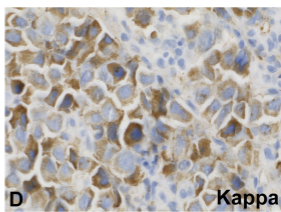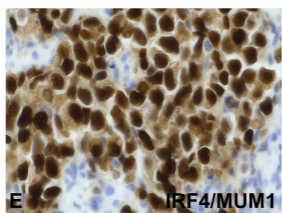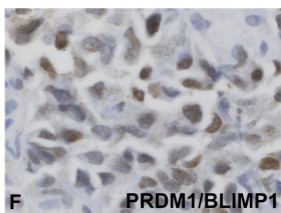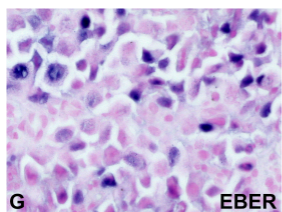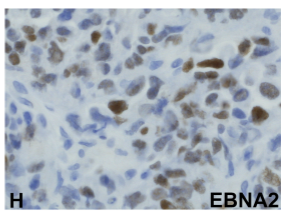

Supplement: Supplementary file 4 — (PDF 5786 kb) [file 10875_2021_1016_MOESM4_ESM.pdf]

Supplemental Figure 2

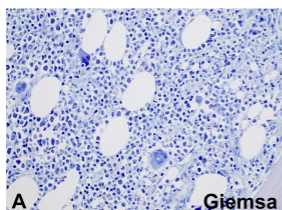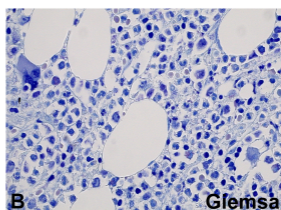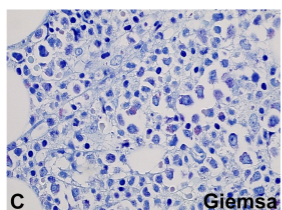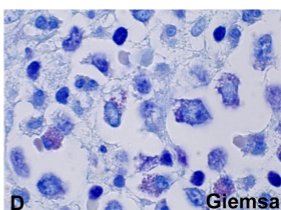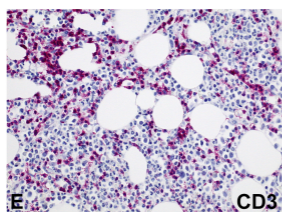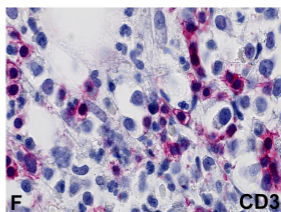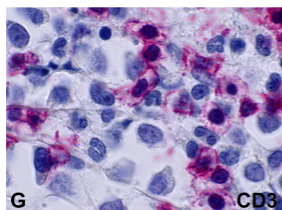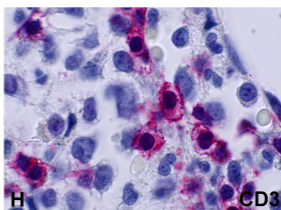

Supplement: Supplementary file 5 — (PDF 6894 kb) [file 10875_2021_1016_MOESM5_ESM.pdf]
